# Natural Agents against Bovine Mastitis Pathogens

**DOI:** 10.3390/antibiotics10020205

**Published:** 2021-02-19

**Authors:** Zorana Kovačević, Miodrag Radinović, Ivana Čabarkapa, Nebojša Kladar, Biljana Božin

**Affiliations:** 1 Department of Veterinary Medicine, Faculty of Agriculture, University of Novi Sad, Trg Dositeja Obradovica 8, 21000 Novi Sad, Serbia; miodrag.radinovic@polj.uns.ac.rs; 2 Institute of Food Technology, University of Novi Sad, Bulevar cara Lazara 1, 21000 Novi Sad, Serbia; ivana.cabarkapa@fins.uns.ac.rs; 3 Center for Medical and Pharmaceutical Investigations and Quality Control/Department of Pharmacy, Faculty of Medicine, University of Novi Sad, Hajduk Veljkova 3, 21000 Novi Sad, Serbia; nebojsa.kladar@mf.uns.ac.rs (N.K.); biljana.bozin@mf.uns.ac.rs (B.B.)

**Keywords:** antibacterial activity, essential oil, mastitis causing bacteria, antioxidant, thymol, antibiotics

## Abstract

Bovine mastitis is the most widespread and economically important disease worldwide. The present study aimed to determine bioactive compounds in two essential oils (EOs) from wild *(Thymus serpyllum)* and common thyme *(Thymus vulgaris)* and to assess the antioxidant potential as well as antibacterial efficacy of the EOs against mastitis-associated bacteria. The study also included antibiotic susceptibility tests. The strains were previously isolated from lactating animals with clinical and subclinical mastitis. The antioxidant potential of the commercial EOs of wild and common thyme was evaluated by five in vitro assays. The antibacterial activity was performed using the microdilution technique, while antibiotic susceptibility testing was performed by the Kirby–Bauer disc diffusion method. The dominant compound in wild thyme was thymol (45.22%), followed by *p*-cymene (23.83%) and γ-terpinene (3.12%), while in common thyme, it was thymol (54.17%), followed by γ-terpinene (22.18%) and *p*-cymene (16.66%). Among the fourteen mastitis-associated bacteria, strain IX *Streptococcus* spp. (β-hemolytic) was the most sensitive to the tested EOs (minimum inhibitory concentration (MIC)/minimal bactericidal concentration (MBC) were 0.78/1.56 and 0.39/0.78 mg/mL for *T. serpyllum* (TS) and *T. vulgaris (TV)*, respectively). Regarding *Streptococcus* spp. β heamoliticus, MICs for *TS* ranged from 0.78 to 1.56 mg/mL, while for the same oil, MBCs ranged from 1.56 to 12.5 mg/mL. In the case of *T. vulgaris,* MICs ranged from 0.39 to 3.125 mg/ mL, while MBCs ranged from 3.125 to 6.25 mg/mL. TV is more active against *E. coli, E. sakazakii,* and *Streptococcus* spp., while it is less effective against *Staphylococcus* spp. than TS. The study revealed that the tested EOs possess remarkable antioxidative and antibacterial activities and could be used in the development of pharmaceutical formulation as an alternative to conventional mastitis therapy.

## 1. Introduction

The economic rise of the dairy market all over the world with the importance of delivering healthy and safety dairy products highlights the importance of managing milk production in a secure and sustainable manner [1,2,3]. In Serbia, milk production is organized in two different systems; small household farms with ten to twenty animals and large farms counting several hundred to several thousand cows [4].

The most common problems influencing animals’ health in both production methods are those related to the health status of the mammary gland [5]. Actually, the occurrence of mastitis is highly frequent and, according to the type of clinical manifestation, this disease has clinical and subclinical classifications that occur simultaneously [6,7]. The etiology of mastitis is complex, and both mechanical and chemical factors that can be attributed to omissions in the way of housing and the procedure of milking cows certainly could contribute to the development of this disease [8]. This problem is more represented on large farms where little human labor is employed. Besides the etiology of mastitis, microbiological factors are more important, dominant bacterial causes [5]. While intramammary administration implies application of antibiotics directly in mammary gland through teat channel in order to achieve their effect locally in the gland, parenteral administration is application where the digestive tract is bypassed (e.g., intramuscularly, subcutaneously, intravenously) in order to achieve systemic effect including mammary gland tissue.

In Serbia, the most common causative agents of mastitis are *Staphylococcus aureus* and *Streptococcus agalactiae*, and recently *E. coli*, while *Klebsiella* spp., coagulase-negative staphylococci and *Streptococcus uberis* are becoming more and more common [5].

The prevalence of bovine mastitis resulted in the extensive use of antibiotics, intramammary and parenterally [9]. Erskine, et al. [10] revealed that 90% of antibiotic residues in milk are a consequence of mastitis therapy. Hence, the use of antibiotics in the treatment of mastitis has some negative consequences, such as entry of antibiotic residues into the human food chain [11], with the possibility of antibiotic-resistant bacteria strains transmission [12]. Additionally, as a negative consequence, increasing resistance of microorganisms to antibiotics causes the degree of intramammary infections cure to be at a very low level. Moreover, the degree of cure in the case of *Staphylococcus aureus* is 20 to 75% [13].

Control of udder health is important for the dairy production chain in light of food safety issues, with control of udder pathogens being the most important in the reduction of foodborne illness and giving healthy dairy food [14,15]. Besides, development and spread of resistance to antibiotics as a consequence of mastitis treatment represent a public health threat to consumers as a global problem, influencing both human and animal health. Faced with the continued growth of antibiotic-resistant pathogens, there is a need for finding novel antimicrobial compounds [16].

Considering the facts mentioned above, many studies tend to develop alternative treatments with bioactive plant-derived products (PDPs), such as plant extracts, essential oils (EOs), hydrolates, oleoresins, and so on [17,18]. Many attempts have been made to test the potential role of EOs and their active compounds to combat antibiotic resistance in bacteria [19].

Furthermore, although many aromatic plants and essential oils are tested, especially for antioxidant and antimicrobial activity, plants belonging to the genus *Thymus*, among others, are of special interest regarding the presence of notable amounts of thymol and carvacrol, being strong antioxidant and antimicrobial agents [20,21,22,23].

Moreover, these studies highlighted a high scientific interest whereby EOs warrant special attention as they are recognized as safe. Besides, EOs do not increase antibiotic resistance during long-term usage, which represents their main advantage [24]. Additionally, synergism between plant metabolites and antibiotics has been described by Hemaiswarya, et al. [25], suggesting the use of EOs as in combination with antibiotics. Furthermore, EOs were characterized by low mammalian toxicity, low environmental effects, and wide public acceptance.

However, it is well known that several chemotypes of *Thymus serpyllum* have been described until now [26,27,28]; as differences in chemical composition have a notable influence on investigated biological activities, new data are of great importance. Besides, it is well known that EOs’ chemical composition, contributing to their medicinal value and being responsible for the biological properties, highly depends on many factors such as geographical and climatic conditions, harvesting, isolation techniques, as well as storage [29,30].

In line with those mentioned above, this study aimed to evaluate the effectiveness of EOs of common (*Thymus vulgaris* L.) and wild thyme (*Thymus serpyllum* L.) against mastitis-associated pathogens in Serbia.

## 2. Results

### 2.1. Bacteriological Testing of Milk Samples

Bacteriological testing was performed on a total of 31 milk samples, while pathogens were isolated in 21 (67.74%) samples. The isolated pathogens were the most common mastitis pathogens, including *Streptococcus* spp. β heamoliticus (Strep_bh), *E. coli* (E_c), *Enterobacter sakazakii* (E_s), *Klebsiella oxytoca* (K_o), *Staphylococcus aureus* (Staph_a), *Staphylococcus* spp. coagulase negative (Staph_cn)*, Streptococcus dysgalactiae* (Strep_d), *Streptococcus* spp. (Strep), and *Streptococcus uberis* (Strep_u).

**Figure 1:**
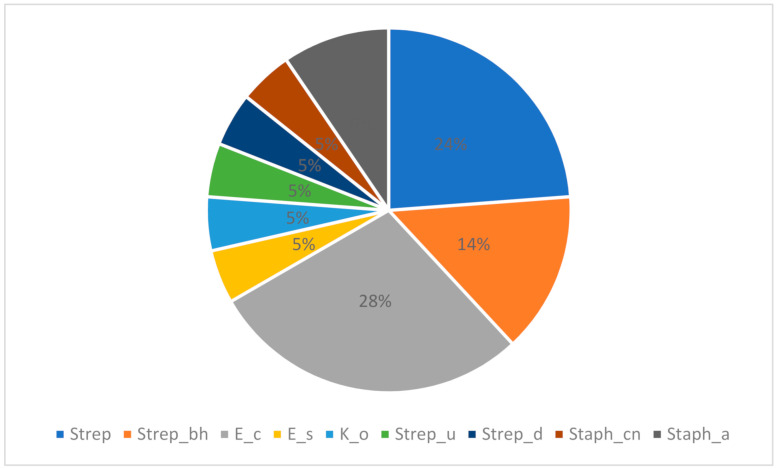
The proportion (%) of the evaluated bacterial strains in the collected samples.

The most common among the pathogens was *E. coli*, which was identified in six samples (19.35%), followed by five (16.13%) samples with *Streptococcus* spp., while *Staphylococcus* spp. coagulase negative, *Streptococcus uberis*, *Streptococcus dysgalactiae, Klebsiella oxytoca*, and *Enterobacter sakazakii* were found in one sample each (3.23%) (Figure 1).

### 2.2. Antibiotic Susceptibility Testing of Mastitis-Associated Bacteria

Antibiotic susceptibility patterns for the analyzed 16 mastitis-associated bacteria are shown in Table 1. Antibiotics included in the testing are amoxycillin (AMX), ampicillin (AMP), ceftriaxone (CRO), enrofloxacin (ENR), erythromycin (ERY), lincomycin (LIN), neomycin (NEO), penicillin (PEN), streptomycin (STR), tetracycline (TET), amoxicillin/clavulanic acid (AMC), novobiocin (NB), trimethoprim/sulfamethoxazole (SXT), and cloxacillin (CLO). In Serbia, the most used antibiotics in mastitis therapy are penicillin, streptomycin, gentamicin, tetracycline, cephalexin, sulfonamides, and enrofloxacin [31].

**Figure 2:**
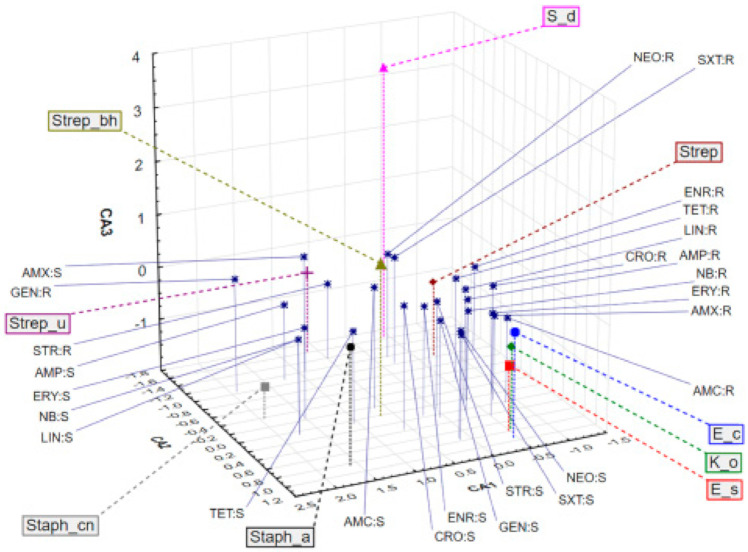
Position of the evaluated bacterial cultures and their sensitivity (S) or resistance (R) to antibiotics treatment in the space defined by the first three correspondent axes.

Application of correspondence analysis (CA) on a dataset describing bactericidal potential of different antibiotics on bacteria isolated from the milk samples shows associations of different bacteria and the evaluated antibiotics in terms of bacteria resistance (R) or sensitivity (S). It must be stated that the results obtained for penicillin (PEN) and cloxacillin (CLO) were not included in statistical processing because of their uniformity. It was observed that the first three correspondent axes (CA1, CA2, and CA3) describe around 50% of the samples’ variability (percentage of inertia for CA1, CA2, and CA3 was 22.14%, 17.83%, and 9.31%, respectively). The position of the evaluated bacteria cultures and antibiotics in the space defined by the first three correspondent axes (Figure 2) shows close association of *E. coli* (E_c), *Klebsiella oxytoca* (K_o) and *Enterobacter sakazakii* (E_s) in the negative part of CA1 and the positive part of CA2 as a result of resistance to erythromycin (ERY), amoxycillin (AMX), and amoxicillin/clavulanic acid (AMC), and sensitivity to neomycin (NEO) and trimethoprim/sulfamethoxazole (SXT). Furthermore, *Streptococcus* spp. (Strep) are localized in the negative part of CA1 and the negative part of CA2, which is closely related to resistance to SXT, NEO, enrofloxacin (ENR), tetracycline (TET), lincomycin (LIN), and ceftriaxone (CRO). *Streptococcus* spp. β-heamoliticus (Strep_bh) are sensitive to AMC, CRO, and ENR. *Staphylococcus aureus* are resistant to ampicillin (AMP), penicillin (PEN), and cloxacillin (CLO).

### 2.3. EOs’ Chemical Composition Analysis

Detailed chemical compositions of the tested wild (*T. serpyllum*) and common thyme (*T. vulgaris*) EOs are listed in Table 2. In the wild thyme EO, there are 19 identified compounds (accounting for 99.29% of total volatile compounds) and, in the common thyme EO, there are 25 compounds (accounting for 99.20% of total volatile compounds). In general, the compounds in both EOs are classified as monoterpenes, with predominance of aromatic oxygenated monoterpenes (51.49% in *T. vulgaris* and 54.98% in *T. serpyllum*). The dominant compounds in *T. serpyllum* EO are thymol (54.17%), γ-terpinene (22.18%), and *p*-cymene (16.66%). In *T. vulgaris* EO, the dominant compounds are thymol (45.22%) and *p*-cymene (23.83%), while the content of γ-terpinene is notably lower (3.12%). *Trans*-β-caryophyllene, a sesquiterpene hydrocarbon, is the third main compound (4.04%) in *T. vulgaris* EO.

### 2.4. EOs’ Antioxidant Potential Evaluation

The antioxidant potential of the tested EOs (*T. vulgaris* and *T. serpyllum*) and the positive control substances were evaluated in a series of in vitro tests (Table 3). All results, except those obtained in the ferric reduction antioxidant potential (FRAP) test, are presented as the inhibitory concentration (IC_50_) values, representing the concentrations of the EOs and positive controls that caused 50% of neutralization, determined by linear regression analysis.

DPPH assay was employed to determine the ability of the tested EOs of common and wild thyme, as well as propyl gallate (PG), to act as donors of hydrogen atoms or electrons in the transformation of DPPH^•^ into its reduced form DPPH-H reaction [32]. Although PG (propyl gallate) (0.71 µg/mL) exhibited very potent free radical scavenging capacity, both EOs were able to reduce the DPPH^•^ into DPPH-H, reaching 50% of reduction (IC_50_ = 16 for *T. serpyllum* and 14 µL/mL for *T. vulgaris*). The free radical scavenging capacity (RSC) of EOs for hydroxyl (OH) radicals was evaluated by measuring the degradation of 2-deoxyribose with OH radicals, generated in the Fenton reaction [32]. Both EOs showed a lower RSC (IC_50_ = 170 for *T. serpyllum* and 230 µg/mL for *T. vulgaris*, respectively) compared with the PG (propyl gallate) (9.07 µg/mL) and *tert-*butylated hydroxytoluene (BHT) (0.03 µg/mL) IC_50_ values, used as standard synthetic antioxidants. However, both of them exhibited protective effects on 2-deoxy-d-ribose degradation, although they were lower compared with ascorbic acid (AA) (2.03 µg/mL). Regarding the neutralization of NO, neither EO reached 50% of reduction. The testing of the ability of the examined EOs to protect the integrity of biological membranes containing lipids was evaluated through determination of lipid peroxidation (LP) inhibition potential, pointing to protective effects of the tested EOs (IC_50_ = 170 for *T. serpyllum* and 190 µg/mL for *T. vulgaris*), but notably lower from those exhibited by BHT (7.29 µg/mL).

The FRAP test showed a notable antioxidant activity for both EOs (29 mg AAE/mL for *T. serpyllum* EO and 34.95 mg AAE/mL for *T. vulgaris* EO).

Comprehensive evaluation of the antioxidant potential in several test-systems showed no significant differences between the examined EOs samples (F (4,1) = 58.82, Wilks = 0.004, *p* = 0.0974).

### 2.5. EOs Effectiveness against Mastitis-Associated Bacteria

EOs effectiveness against mastitis-associated bacteria was expressed as minimum inhibitory concentrations (MICs) and minimal bactericidal concentrations (MBCs) (Table 4). Among the fourteen mastitis-associated bacteria, strain IX *Streptococcus* spp. (β-hemolytic) showed the most sensitivity to the tested EOs (MIC/MBC were 0.78/1.56 and 0.39/0.78 mg/mL for *T. serpyllum* and *T. vulgaris*, respectively). Regarding *Streptococcus* spp. β heamoliticus, MICs for *T. serpyllum* ranged from 0.78 to 1.56 mg/mL, while for the same oil, MBCs ranged from 1.56 to 12.5 mg/mL. In the case of *T. vulgaris,* MICs ranged from 0.39 to 3.125 mg/ mL, while MBCs ranged from 3.125 to 6.25 mg/mL. TV is more active against *E. coli*, *E. sakazakii,* and *Streptococcus* spp., while it is less effective against *Staphylococcus* spp. than TS.

### 2.6. Interpretations of MBC, MIC, *Thymus vulgaris*, and *Thymus serpyllum* EOs in Relation to the Chemical Composition of the EOs

**Figure 3:**
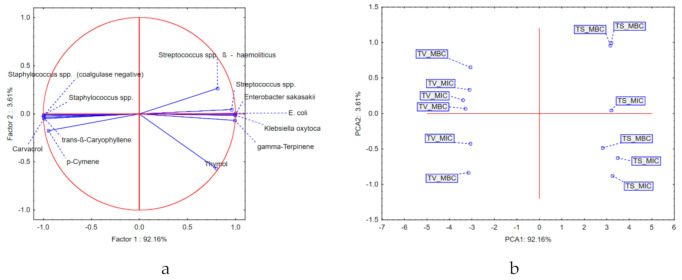
Results of principal components analysis (PCA): (**a**) loadings of the first two principal components; (**b**) position of the examined samples in the space defined by the first two principal components. TS, *T. serpyllum*; TV, *T. vulgaris*; MIC, minimum inhibitory concentration; MBC, minimal bactericidal concentration.

Application of principal components analysis (PCA) on the results describing the MIC and MBC of the tested EOs in relation to the most abundant secondary metabolites showed that the first two principal components (PCs) describe more than 95% of the dataset variability, while the principal components axis 1 (PCA1) describes more than 90% of the samples’ variability. It can be observed that most of the variability is explained by the results describing the antibacterial potential in the case of *Streptococcus* spp., *Enterobacter sakazakii*, *Klebsiella oxytoca*, and *Staphylococcus* spp., as well as by the chemical composition in terms of carvacrol, trans-β-caryophyllene, and γ-terpinene content. Positioning of *Thymus vulgaris* EO samples in the negative part of PCA1 suggests that these samples, in relation to *Thymus serpyllum* EO, are characterized by the presence of significant amounts of carvacrol, trans-β-caryophyllene, and p-cymene. Furthermore, the thyme EO showed stronger antibacterial potential against *Streptococcus* spp. (β-hemolytic), *Streptococcus* spp. *Enterobacter sakazakii*, *E. coli*, and *Klebsiella oxytoca* in comparison with the wild thyme EO. On the other hand, *Thymus serpyllum* EO samples grouped in the positive part of PCA1 (as a result of higher amounts of thymol and γ-terpinen), and thus showed stronger antibacterial activity against *Staphylococcus* spp. (coagulase-negative) and *Staphylococcus* spp. (Figure 3).

## 3. Discussion

Considering the importance of the issue of antimicrobial resistance (AMR), scientists are trying to find an alternative to antibiotics in mastitis therapy [33,34,35]. Recently, phytotherapy has been finding a place in the vast drug market owing to its capacity to prevent the development and spread of AMR. Phytotherapy in this way also reduces the economic losses in the dairy industry caused by rejection of milk due to antibiotic withdrawal time [36,37].

In order to develop an EO-based pharmaceutical formulation, it is necessary to perform chemical composition analysis and testing of EOs against the most common mastitis pathogens.

Analysis of EOs’ chemical composition in this study revealed that both tested essential oils are in accordance with the requirements prescribed by Ph. Eur. 10 (2020) [38] for thymol type of thyme EOs, with thymol ranging from 37 to 55% and carvacrol from 0.5 to 5.5%. According to the pharmacopoeia, the official biological source of *Thymus vulgaris* EO is only the flowering aerial parts of *Thymus vulgaris*, *T. zygis*, or a mixture of both species, while *T. serpyllum* is the biological source of *Serpylli herba*. However, the content of thymol in the wild thyme EO is higher (54.17%) than in the common thyme EO (45.22%). Unlike thymol, the content of carvacrol is reversed (3.86% in common thyme and 0.81% in wild thyme). There is a notable difference between the tested EOs in the content of *p*-cymene in common thyme (23.83%) and wild thyme (16.66%), but both EOs meet the quality criteria prescribed by the pharmacopoeia (14–28%). On the other hand, the content of γ-terpinene (3.12% for common thyme and 22.18% for wild thyme) does not comply with the pharmacopoeia requirements (4–12%). According to the prescribed content of linalool (1.5–6.5%) and terpinen-4-ol (0.1–2.5%) established by the pharmacopoeia, *T. vulgaris* EO meets the requirements related to the chemical composition for thymol type EO (2.55% for linalool and 1.42% for terpinen-4-ol). The results for *T. vulgaris* EO composition are also in line with previously published data [20,21,39]. However, because, in the wild thyme EO, several chemotypes are described [26,27,28], this oil could be defined as a thymol chemotype.

However, the chemical composition of *T. vulgaris* EO of commercial origin [40,41], and from different regions [36,37,38] showed different thymol chemotypes, with the thymol being the more abundant compound.

It is well known that plants possess significant antioxidant potential, mainly attributed to the presence of different aromatic, phenolic, and flavonoid compounds [42]. Nowadays, the trends in food and cosmetic industry suggest the use of natural products, especially as a replacement for synthetic antioxidants [43]. Although EOs in all of the tested systems exhibited weaker free radical scavenging effects, it must be emphasized that the comparison of the antioxidant potential in the present study was performed between pure compounds and EOs, which are mixtures of different secondary metabolites, meaning that some of them do not possess potential for scavenging reactive oxygen species (ROS) and preventing biological membrane degradation. Regarding their toxicity, synthetic antioxidants are abused in some food and cosmetic products, especially *tert-*butylated hydroxytoluene (BHT) [44].

Regarding that fact, the EOs tested in our study showed notable antioxidant potential, similar to the results of other authors [20,21,27,28,32,45], although the general comparison of the results obtained in different labs is sometimes difficult because of different experimental conditions and presentation of the results, different methods of antioxidant potential evaluation, and so on. In addition, in the case of various *Thymus* species EOs, the chemical composition plays a significant role in antioxidant effects as thymol and carvacrol are the main compounds responsible for RSC and inhibition of peroxidation of different lipids and biological membranes [20,21]. Both thymol and carvacrol demonstrate the ability to achieve a resonantly stable radical structure after donation of hydrogen atom or electrons to ROS, thus neutralizing the cascade of free radical reactions [42]. Hence, different chemotypes, especially of different species of wild thyme (*T. serpyllum*, *T. marschallianus*, *T. jankae*, *T. longicaulis*, *T. lonidens*, *T. pannonicus,* and so on), can show significantly weaker antioxidant effects [27]. As different plant-derived products have less toxicity and side effects compared with synthetic antioxidants, they can play an important role in the prevention of various diseases and syndromes where reactive oxygen species are involved [42]. They have also found their place as natural preservatives in the pharmaceutical, food, and cosmetic industry [43]. ROS production is linked with the inflammatory process and is provided by netrophils in milk [46].

The results obtained in this study indicated that the common thyme EO showed stronger antibacterial potential against *Streptococcus* spp. (β-hemolytic), *Streptococcus* spp. *Enterobacter sakazakii*, *E. coli*, and *Klebsiella oxytoca* in comparison with the wild thyme EO. On the other hand, wild thyme EO samples grouped in the positive part of PCA1 (as a result of higher amounts of thymol and γ-terpinen), and thus showed stronger antibacterial activity against *Staphylococcus* spp. (coagulase-negative) and *Staphylococcus* spp. (Figure 3). The significant antimicrobial activity of *T. vulgaris* EO against *Staphylococcus* spp. isolated from bovine mastitis has been previously confirmed [47,48]. These studies reported phenolic compounds such as carvacrol and thymol as major constituents of the tested EO are responsible for their antimicrobial activities.

Phenolic compounds (carvacrol and thymol) account for 54.98% of the total oil in *T. serpyllum* EO and 49.08% in *T. vulgaris* EO. The antibacterial effect of the EOs tested in our study probably depends on these compounds, and a number of studies revealed that phenolic compounds, such as carvacrol and thymol, possess antibacterial activity [21,49,50,51]. Furthermore, the main constituents of the EOs tested in our study are monoterpenes (thymol, carvacrol, p-cymene, and γ-terpinene), which showed a remarkable inhibitory effect against different pathogens such as *Staphylococcus aureus, Escherichia coli* O157:H7, *Salmonella Infantis*, *Bacillus cereus*, and *Clostridium perfringes* [21,50,52]. From the chemical point of view, carvacrol and thymol represent structural isomers and possess differently located phenolic hydroxyl on the phenolic ring [53]. Some studies indicated that the hydroxyl group has a part in increasing their hydrophilic ability, helping them to dissolve in the microbial membrane and impair them [54,55,56,57].

Compared with carvacrol, thymol also possesses similar antibacterial activity, even though its hydroxyl group is located in a different position [58]. The thymol primary mode of antibacterial action is partly understood and it is probably similar to carvacrol. This mode of action results in structural and functional alterations in the cytoplasmic membrane that can damage the outer and inner membranes and interact with membrane proteins, as well as intracellular targets [59]. In contact with the cell membrane, thymol can modify membrane permeability, leading to the release of K^+^ ions and ATP [54,60,61]. Some studies revealed that thymol integrates within the polar head-groups of the lipid bilayer, causing alterations of the cell membrane [54,60,62]. In contrast to the efficiency of monoterpenes with added oxygen molecules (carvacrol and thymol), monoterpene hydrocarbons p-cymene and γ-terpinene used separately do not demonstrate remarkable inhibitory effects against bacteria growth [50,63].

However, several studies indicated that *p*-cymene can enhance the inhibitory effects of carvacrol when these two compounds are used together [58,64,65]. It was also shown that *p*-cymene, owing to its hydrophobic nature, greatly contributes to the cytoplasmic membrane swelling [61]. The findings obtained by Ultee, Bennik, and Moezelaar [61] indicated that *p*-cymene enabled carvacrol to be more easily transported into the cell. With respect to these findings, a slightly higher antibacterial effect of *T. vulgaris* EO obtained in this research could be accounted for by a slightly higher content of *p*-cymene.

Fratini et al., examining the efficiency of *Thymus vulgaris* L. ct. carvacrol and *T. vulgaris* L. ct. thymol), two selected mixtures of EOs, and two artificial mixtures of their main constituents (thymol, carvacrol, and p-cymene), against the bacterial strains involved in the pathogenesis of mastitis using the Kirby–Bauer method, confirmed that thymol and carvacrol as main constituents of tested EO are responsible for antibacterial activity. Moreover, the highest antibacterial effectiveness was obtained with the artificial mixture of pure constituents (carvacrol and thimol) with the addition of p-cymene [48].

This study revealed significant resistance of the most common mastitis pathogens to antimicrobials, indicating the importance of finding an alternative to antibiotic treatment in therapy of this disease. Moreover, it was shown that gram-negative pathogens (*E. coli*, *Klebsiella oxytoca* and *Enterobacter sakazakii*) are resistant to erythromycin, amoxycillin, and amoxicillin/clavulanic acid. This is in accordance with results of other studies, which determined resistance to the mentioned antimicrobials [66]. In addition, *Streptococcus* spp. isolated in this study showed resistance to trimethoprim/sulfamethoxazole, neomycin, enrofloxacin, tetracycline, lincomycin, and ceftriaxone, which is in line with the resistance determined in other studies [67].

Interestingly, although studies conducted so far highlighted the possibilities of EOs as a potential resistance-modifying agent, they provided limited evidence suggesting the spontaneous occurrence of resistance to EOs [68]. In fact, resistance of bacteria to EOs and their active components depends on their chemical composition, as well as their mechanism of action, which is specific and completely different compared with antibiotics. The antimicrobial activity of these natural substances has not been fully studied, but, owing to the proven ability of some EOs to inhibit bacterial cell wall synthesis, block transcription, β-lactamase production, biofilm formation, or efflux pump operation, they are considered to be useful in the treatment of infections caused by resistant microorganisms [68,69]. Besides, the difference between the mechanisms of resistance in EOs and antimicrobials is giving immense potential for the replacement of conventional antimicrobial therapy with phytotherapy.

## 4. Material and Methods

### 4.1. Essential Oils

In the present study, the essential oils of common (*Thymus vulgaris* L., Lamiaceae) and wild thyme (*Thymus serpyllum* L., Lamiaceae), commercially available on the Serbian market and produced by a certified manufacturer (Pharmanais d.o.o., Serbia), were evaluated in the study. Row plant material (*Thymi folium* and *Serpylli herba*) was sampled before distillation from the manufacturer and confirmed for identity. Voucher specimens (Tv-03/2020 and Ts-2/2020, respectivelly) were deposited at the Herbarium of drugs of the Pharmacognosy and phytotherapy laboratory, Department of Pharmacy, Faculty of Medicine, University of Novi Sad. According to the certificate obtained from the manufacturer for both samples, essential oils were obtained using the internal steam distillation technique (Cellkraft AB, Sweden).

### 4.2. Sampling Procedure

The milk samples were collected from four Holstein-Friesian dairy farms located in Serbia. The number of cows on the farms varied, ranging from twenty to three hundred. The samples were taken from lactating animals with clinical and subclinical mastitis, without other health problems. Clinical mastitis was diagnosed by clinical examination of udder, while subclinical mastitis was confirmed using somatic cell count in the milk samples.

Bacteriological testing was performed by taking aseptic milk samples from all animals (clinical and subclinical mastitis) during the morning milking. The samples were then taken in sterile tubes marked with an ID number of the cow and stored at 4 °C. Afterwards, the samples were processed at the Laboratory for Milk Hygiene at the Department of Veterinary Medicine, Faculty of Agriculture, University of Novi Sad. The samples were inoculated on 2% blood agar, using a platinum loop (0.01 mL), followed by incubation of the samples for 48 h at 37 °C. Biochemical and cultural characteristics of the grown microorganisms were taken into account during their determination. Isolation and identification of bacterial strain from milk samples was conducted using microbiological procedures for the diagnosis of udder infection published by National Mastitis Council. A loopful of milk sample was streaked on blood agar (Oxoid) and then subcultured on the following selective media: Mannitol Salt Agar, Edwards Agar, Salmonella-Shigella Agar, and MacConkey Agar. Then, plates were incubated aerobically at 37 °C for 24 h. After incubation, the plates were examined for colony morphology, pigmentation, and hemolytic characteristics at 24–48 h. Catalase test was applied for distinguishing between staphylococci and other Gram-positive cocci, mannitol fermentation test, coagulase test (either positive or negative), hemolytic pattern, and colony morphology. The isolates were confirmed by biochemical tests: oxidase activity, acid production (lactose sucrose and glucose fermentation), indole production, Voges–Proskauer, and hydrogen sulfide production. In addition, each strain was confirmed using Analytical Profile Index API-20 tests (API, bio Meraux, France). To isolate staphylococci, listed media were used: blood agar, nutrient agar, Ziehl–Neelsen, MSA, for *E. coli* isolation nutrient agar, MacConkey agar, and API 25 were used. For streptococci, Edwards agar and esculin were used. Of the phenotypic characteristics for staphylococci, the occurrence of α and β hemolysis and, for E. coli, there were pink colonies with precipitation. For streptococci determination, hydrolysis of esculin was used.

### 4.3. Antibiotic Susceptibility Testing of Mastitis-Associated Bacteria

The antibiotic susceptibility patterns for the 16 mastitis-associated bacteria were established in vitro, following the Kirby–Bauer disc diffusion method, on Mueller–Hinton agar (Oxoid) [70]. Antibiotic susceptibility testing was conducted using commercially available antibiotic disks (Bioanalyse) in the following concentrations: ampicillin (10 µg); streptomycin (10 µg); gentamicin (10 µg); trimethoprim/sulphamethoxazole (1.25/23.75 µg); enrofloxacin (5 µg); and ceftriaxone (30 µg). The isolates and reference strains were inoculated on nutrient broth separately and incubated aerobically at 37 °C. After overnight incubation, the bacterial suspension was vortexed and diluted to a turbidity equivalent to that of 0.5 McFarland standards. The bacterial suspension was then spread onto the surface of the Mueller–Hinton agar to make confluent growth. Antibiotic discs were immediately placed on the surface of the agar plate using forceps and incubated aerobically at 37 °C for 16 h. Inhibition zones for various isolates were measured and interpreted as sensitive, intermediate, or resistant according to the Clinical Laboratory Standards Institute (CLSI) [71,72].

### 4.4. EOs Chemical Composition Analysis

The qualitative and quantitative analysis of the EOs was carried out on HP-5MS capillary column (30 m × 0.25 mm; film thickness 0.25 μm) on Agilent 6890B gas chromatograph coupled with flame ionization detector (GC-FID) instrument coupled to Agilent 5977 mass spectrometry detector (MSD) (Agilent Technologies Inc, Santa Clara, CA, USA, USA). The samples were injected in split mode 1:20, at an inlet temperature of 220 °C. The oven temperature was set at 60 °C and increased at a rate of 3 °C/min up to 246 °C. Helium was the carrier gas (1 mL/min), while the temperature of the MSD transfer line was set to 230 °C.

Mass spectral data were collected in scan mode (*m/z* = 50–550), while the identification of compounds was performed using NIST (v14, National Institute of Standards and Technology, Gaithersburg, MD, USA) mass spectral database and comparison of relative retention indices (RT), as well as literature data [73].

### 4.5. EOs’ Antioxidant Potential Evaluation

The antioxidant potential of the commercial EOs of wild (*Thymus serpyllum*) and common thyme (*Thymus vulgaris*) was evaluated in five in vitro assays, as single models are not recommended for evaluation because of the complex composition of different plant extracts [74]. The potential of the EOs to neutralize 2,2-diphenyl-1-picrylhydrazyl (DPPH), hydroxyl (OH) and nitroso (NO) radicals was assessed by previously described spectrophotometric methods [32]. Moreover, the ability of the examined EOs to inhibit the processes of lipid peroxidation (LP) is evaluated, using liposomes emulsion as a test model [20]. The potential of the examined EOs to reduce Fe^3+^ (ferric reduction antioxidant potential—FRAP test) was assessed by the method described by Lesjak et al. [45], as it is a model correlating with the neutralization of hypochlorite and peroxynitrite anion [75]. The results obtained in the FRAP assay were expressed as ascorbic acid equivalents (AAEs) based on the previously constructed calibration curve for ascorbic acid. For each sample, four replicates were recorded in all test-systems. Synthetic antioxidants, including ascorbic acid (AA), propyl gallate (PG), and *tert-*butylated hydroxytoluene (BHT), were tested under the same experimental conditions as positive control for antioxidant potential of the tested EOs.

### 4.6. EOs’ Effectiveness Determination against Mastitis-Associated Bacteria

EOs’ effectiveness on planktonically grown bacteria was determined according to the Clinical Laboratory Standards [76] with slight modifications. The bacterial suspensions were prepared using overnight cultures and adjusted to 0.5 Mc Farland standard turbidity (corresponding to 1 × 10^8^ CFU/mL), using a densitometer DEN-1 (Biosan, Riga, Latvia). All tests were performed in Muller–Hinton broth (MHB) (Lab M, International Diagnostics Group Plc, Bury, Lancashire, UK). MHB supplemented with 0.5% Tween 80 (Polyoxyethylenesorbitan monooleate, HiMedia Laboratories Pvt. Ltd., Mumbai, India) was used for dissolving the EOs, as well as for their dilution to the concentration ranging from 1000 to 0.9 mg/mL. Twenty-microliter aliquots of each tested EO were added to 96-well microtiter plates. Afterward, aliquots of 160 µL of MHB were added to each well. As the final step, 20 µL of the standardized bacterial suspension was inoculated into each well. The test was performed in a total volume of 200 µL with final EOs’ concentrations ranging from 100 to 0.09 mg/mL, while the final microbial concentration was 10^7^ CFU/mL. The plates were incubated at 37 °C for 24 h. The same tests were performed simultaneously for growth control (MHB + test organism), negative control (MHB + solvent + test organism), and sterility control (MHB + test oil).

Following the incubation, 10 µL of the resazurin solution (0.01%) (Sigma-Aldrich, St Louis, MO, USA) was added to each well. Subsequently, the plates were further incubated at 37 °C for 6 h (in darkness). After visual examination, the plates were additionally incubated for 18 h. Change of color from blue (oxidized) to pink (reduced) indicated the growth of bacteria. On completion of the incubation, wells without the color change (blue color of resazurin remained unchanged) were scored as above the minimum inhibitory concentration (MIC) value. MIC was defined as the lowest concentration at which the color change occurred [77,78].

Referring to the results of the MIC assay, the wells showing complete absence of growth were identified and 100 µL of the solutions from each well was transferred to plate count agar plates (PCA) (Lab M, International Diagnostics Group Plc, Bury, Lancashire, UK) and incubated at 37 °C for 24 h. The minimal bactericidal concentration (MBC) was defined as the lowest concentration of the EOs at which 99.9% of the inoculated bacteria were killed.

### 4.7. Data Analysis

The results obtained in the study were processes by MS Office Excel v2019 (Microsoft Corporation, Redmond, WA, USA) and Statsoft Statistica v12.5 (Hamburg, Germany) software. The values were expressed as the mean values corrected by standard deviation (SD). Methods of univariate and multivariate statistical analysis (MANOVA, pincipal component analysis (PCA) and correspondence analysis (CA)) were applied for comprehensive evaluation of the relations in the obtained dataset.

## 5. Conclusions

This study highlighted that two thyme (*Thymus serpyllum* and *T. vulgaris*) EOs can be used in the development of pharmaceutical formulation as an alternative to conventional mastitis therapy, owing to the EOs’ chemical composition, antioxidant potential, and effectiveness against mastitis-associated bacteria. Further research on dairy farms is needed to conduct clinical trials of EO-based formulation. Moreover, additional studies should explore their toxicity to mammalian cells and drug-like properties (pharmacokinetic and pharmacodynamic) to determine their potential as therapeutic agents. Finally, further studies also need to compare the economic aspects of the conventional versus herbal treatment of mastitis, as well as the combination of EOs with common antibiotics used in the treatment of mastitis.

## Figures and Tables

**Table 1:** Antibiotic susceptibility patterns for the mastitis-associated bacteria (S—sensitive, I—intermediate, R—resistant). AMX, moxycillin; AMP, ampicillin; CRO, ceftriaxone; ENR, enrofloxacin; ERY, erythromycin; LIN, lincomycin; NEO, neomycin; PEN, penicillin; STR, streptomycin; TET, tetracycline; AMC, amoxicillin/clavulanic acid; NB, novobiocin; SXT, trimethoprim/sulfamethoxazole; CLO, cloxacillin.

Bacterial Strains Culture	AMX	AMP	CRO	ENR	ERY	GEN	LIN	NEO	PEN	STR	TET	AMC	NB	SXT	CLO
*Streptococcus* spp. β heamoliticus	S	S	S	S	S	R	S	R	R	S	S	S	S	R	R
*Staphylococcus* spp.	R	R	R	R	R	I	R	R	R	S	R	R	R	R	R
*Staphylococcus* spp.	R	R	R	R	R	I	R	R	R	S	R	R	R	R	R
*Staphylococcus* spp. coagulase negative	S	S	I	S	S	S	S	S	R	S	S	S	S	S	R
*Staphylococcus* spp.	R	R	R	R	I	S	R	R	R	S	R	R	R	R	R
*Streptococcus* spp. β heamoliticus	I	R	S	S	R	S	R	S	R	S	I	S	I	S	R
*E. coli*	R	R	R	S	R	S	R	S	R	S	R	R	R	S	R
*E. coli*	R	R	R	S	R	S	R	S	R	S	I	R	R	S	R
*Streptococcus* spp. β heamoliticus	R	R	S	S	R	S	R	S	R	S	R	S	R	R	R
*Klebsiella oxytoca*	R	R	S	S	R	S	R	S	R	S	R	R	R	S	R
*E. coli*	R	R	R	S	R	S	R	S	R	S	I	R	R	S	R
*Staphylococcus* spp.	R	R	I	S	R	S	I	R	R	S	R	S	R	R	R
*E. coli*	R	R	R	S	R	S	R	S	R	S	R	R	R	S	R
*Enterobacter sakazakii*	R	R	R	S	R	S	R	S	R	S	S	R	R	S	R
*Staphylococcus aureus*	I	R	S	S	S	S	S	S	R	S	S	S	S	S	R
*E. coli*	I	R	S	S	R	S	R	S	R	S	I	S	R	S	R
*Streptococcus uberis*	S	S	I	S	I	S	R	R	R	S	S	S	R	R	R
*E. coli*	I	R	S	S	R	S	R	S	R	S	S	S	R	S	R
*Staphylococcus aureus*	I	R	S	S	S	S	S	S	R	S	S	S	S	S	R
*Streptococcus dysgalactiae*	S	R	R	S	I	I	R	R	R	S	R	S	I	R	R
*Staphylococcus* spp.	S	S	S	S	S	S	R	R	R	R	R	S	R	R	R

**Table 2:** Chemical composition of *T. serpyllum* and *T. vulgaris* essential oils (EOs) (%).

Peak No.	Compounds	RI ^a^	*T. vulgaris*	*T. serpyllum*
Monoterpene Hydrocarbons			10.84	25.4
**1.**	α-Pinene	937	1.51	0.18
**2.**	Camphene	952	1.67	0.19
**3.**	β-Pinene	978	0.21	2.15
**4.**	β-Myrcene	991	1.64	0.28
**5.**	α-Phellandrene	1005	0.11	0.08
**6.**	α-Terpinene	1017	0.87	0.13
**8.**	Limonene	1030	1.71	0.21
**12.**	γ-Terpinene	1060	3.12	22.18
**Aromatic Monoterpene Hydrocarbons**			**23.83**	**16.66**
**7.**	p-Cymene	1025	23.83	16.66
**Oxygenated Monoterpenes**			**7.19**	**2.05**
**9.**	1,8-Cineole	1032	0.93	0.16
**10.**	Linalool	1099	2.55	0
**11.**	Camphor	1145	0.33	0.77
**13.**	endo-Borneol	1167	1.68	0
**14.**	Terpinen-4-ol	1177	1.42	0.07
**15.**	Isomenthol	1183	0	0.84
**16.**	α-Terpineol	1189	0.23	0.03
**19.**	Bornyl acetate	1285	0.05	0.07
**26.**	trans-β-Ionone	1486	0	0.11
**Aromatic Oxygenated Monoterpenes**			**51.49**	**54.98**
**17.**	Isothymol methyl ether	1230	0.92	0
**18.**	Thymol methyl ether	1235	1.49	0
**20.**	Thymol	1291	45.22	54.17
**21.**	Carvacrol	1299	3.86	0.81
**Sesquiterpene Hydrocarbons**			**4.91**	**0.2**
**22.**	α-Cubebene	1351	0.08	0
**23.**	β-Cubenene	1388	0.03	0
**24.**	trans-β-Caryophyllene	1419	4.04	0.12
**25.**	Humulene	1454	0.41	0.08
**27.**	δ-Cadinene	1524	0.35	0
**Oxygenated Sesquiterpenes**			**0.94**	**0**
**28.**	Caryophyllene oxide	1581	0.94	0
**Total of identified compounds (%)**			**99.2**	**99.29**

^a^ Retention indices (RI) relative to C9–C24 n-alkanes on the HP 5MS column.

**Table 3:** Antioxidant potential of the investigated essential oils of *T. serpyllum* and *T. vulgaris* and positive control substances (**AA**—ascorbic acid; **PG**—propyl gallate; **BHT**—*tert-*butylated hydroxytoluene). FRAP, ferric reduction antioxidant potential; DPPH, 2,2-diphenyl-1-picrylhydrazyl; OH, hydroxyl; LP, lipid peroxidation.

Samples	Assay			
	DPPH IC_50_	OH IC_50_ (µg / mL)	LP IC_50_	FRAP (mg AAE ^a^ /mL EO)
	X̅ ^b^ ± SD ^c^	X̅ ± SD	X̅ ± SD	X̅ ± SD
***T. vulgaris***	14 ± 0.85	230 ± 1.19	19 ± 1.02	34.95 ± 3.50
***T. serpyllum***	16 ± 0.93	170 ± 2.02	17 ± 0.92	29.00 ± 2.90
**AA**	/	2003 ± 0.39	/	/
**PG**	0.71 ± 0.04	9.07 ± 0.59	/	/
**BHT**	/	0.03 ± 0.01	7.29 ± 0.56	/

^a^ ascorbic acid equivalents; ^b^ mean value; ^c^ standard deviation.

**Table 4:** Minimum inhibitory concentrations (MICs) and minimal bactericidal concentrations (MBCs) of *T. serpyllum* and *T. vulgaris* EOs against mastitis-associated pathogens.

Sample	TV * (MIC) (mg/mL)	TV * (MBC) (mg/mL)	TS ** (MIC) (mg/mL)	TS ** (MBC) (mg/mL)
4 *E. coli*	3.125	6.25	6.25	12.5
*Enterobacter sakazakii*	3.125	6.25	6.25	12.5
*Streptococcus* spp. β heamoliticus	0.39	0.78	1.56	3.125
*Streptococcus* spp. β heamoliticus	0.78	1.56	1.56	3.125
*Streptococcus* spp. β heamoliticus	0.39	0.78	0.78	1.56
*Streptococcus* spp.	1.56	3.125	3.125	6.25
*Streptococcus* spp.	0.78	1.56	3.125	6.25
*Streptococcus* spp.	1.56	6.25	3.125	6.25
*Staphylococcus* spp.	6.25	12.5	3.125	6.25
*Staphylococcus* spp. coagulase negative	6.25	12.5	3.125	6.25
*Klebsiella oxytoca*	1.56	6.25	3.125	6.25

* T. serpyllum (TS) EO. ** T. vulgaris (TV) EO.

## Data Availability

Data is contained within the article.
